# T-cell metabolic ageing as a factor for therapeutic research

**DOI:** 10.1093/cei/uxag066

**Published:** 2026-10-27

**Authors:** Ellen King, Bahar Zinali Sohanaki, Anna Schurich

**Affiliations:** Department of Infectious Diseases, School of Immunology and Microbial Sciences, King’s College London, London, UK; Department of Infectious Diseases, School of Immunology and Microbial Sciences, King’s College London, London, UK; Department of Infectious Diseases, School of Immunology and Microbial Sciences, King’s College London, London, UK

**Keywords:** T cell, metabolism, ageing

## Abstract

Ageing is an important determinant of immune function across the lifespan, yet it remains underappreciated in the design and interpretation of much basic T-cell research. As the immune system ages, T-cell responses become less diverse, less efficient, and less adaptable, with consequences for infection control, vaccine responsiveness, cancer immunosurveillance, and the performance of T-cell-based immunotherapies. This issue is particularly acute because many fundamental studies still rely on T cells from, mostly, healthy young donors. These controls are indispensable, but the field has built much of its pre-clinical logic on immune systems that are younger, fitter, and biologically distinct from the patients for whom T-cell therapies are intended. We argue that this mismatch between experimental systems and clinical reality could be overcome by considering age as a routine variable from the start.

## Teaser

The T cell pool shifts from highly diverse naïve cells expressing CD45RA, CCR7 and CD62L in early life toward increasingly abundant memory and effector cells, with greater glycolytic activity during effector function and FAO/OXPHOS reliance in memory cells. In later life, naïve cells decline while chronic antigen exposure promotes late-differentiated and exhausted/senescent phenotypes, marked by KLRG1, CD57, PD-1, LAG-3 and TIM-3, contributing to impaired vaccine responses and infection susceptibility.

## Graphical Abstract

**Graphical Abstract:**
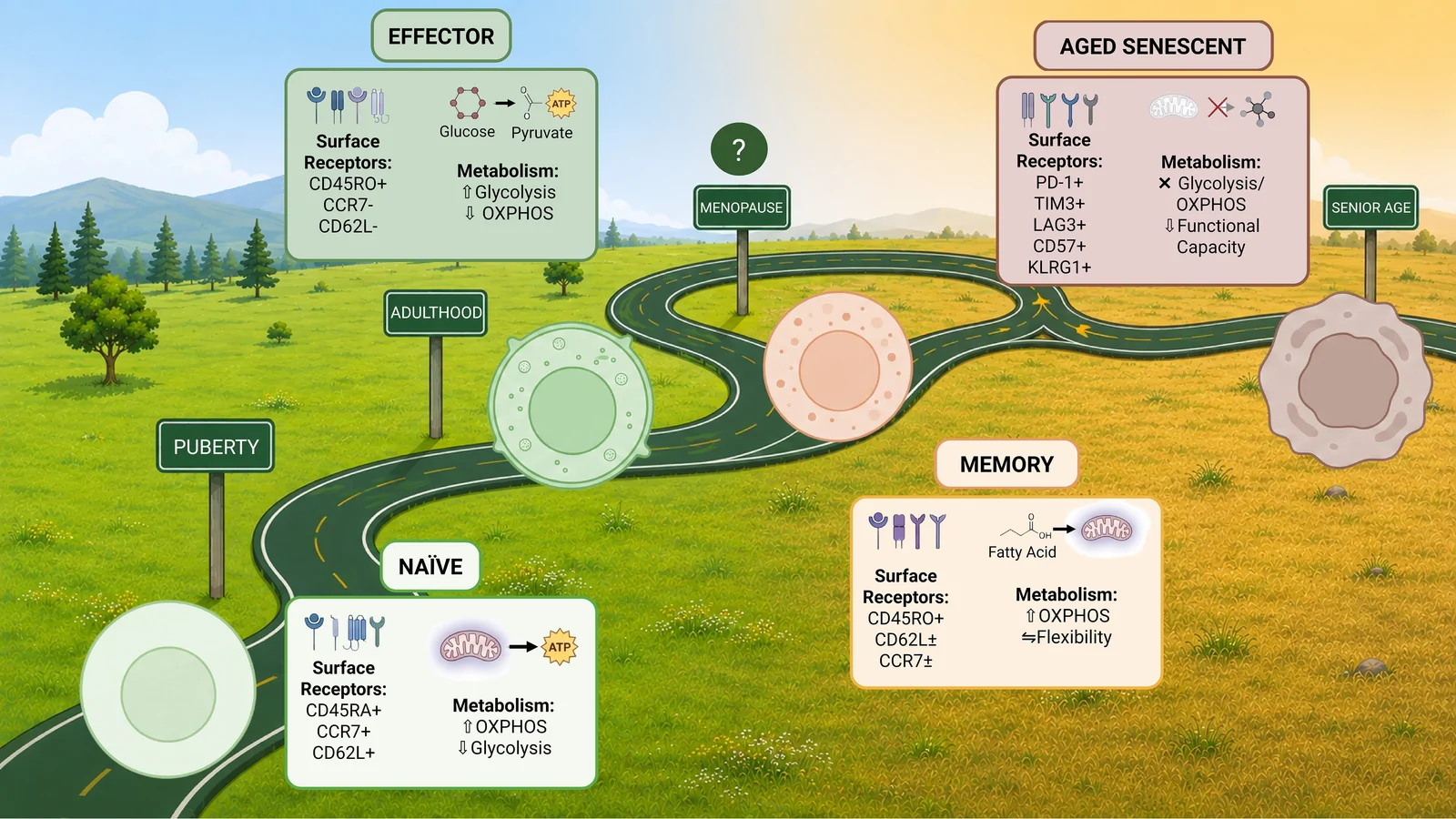
How the T-cell milieu ages. The T-cell pool shifts from highly diverse naïve cells expressing CD45RA, CCR7 and CD62L in early life toward increasingly abundant memory and effector cells, with greater glycolytic activity during effector function and FAO/OXPHOS reliance in memory cells. In later life, naïve cells decline while chronic antigen exposure promotes late-differentiated and exhausted/senescent phenotypes, marked by KLRG1, CD57, PD-1, LAG-3 and TIM-3, contributing to impaired vaccine responses and infection susceptibility.

## Introduction

The global population is undergoing a significant demographic shift towards an older age structure, with projections suggesting that by the mid-21st century, approximately one quarter of individuals will be over the age of 65 [1]. Ageing is strongly associated with the development of multiple comorbidities, including metabolic disorders, autoimmune conditions, neurological decline, and an increased vulnerability to infections. In addition, advancing age remains one of the strongest risk factors for cancer [2]. A key feature of the ageing process is the progressive dysregulation of the immune system, which is known as immunosenescence. T cells play a central role in adaptive immunity; however, ageing significantly alters their phenotype, function, and metabolic state, contributing to the altered immune responses observed in older individuals.

These changes are shaped by both intrinsic ageing processes and lifetime immune experience. Older individuals have been exposed to more infections, vaccinations, inflammatory insults, and environmental challenges than younger individuals. At the same time, thymic involution progressively reduces the generation of new naïve T cells. As a consequence, ageing is associated with reduced T-cell receptor repertoire diversity, a decline in naïve T cells and a corresponding accumulation of memory and terminally differentiated populations, particularly well described within the CD8+ compartment [2]. This shift is accompanied by reduced proliferative capacity and the increased appearance of phenotypes associated with exhaustion and senescence [3]. Collectively, these changes reduce the ability of the aged immune system to respond to new challenges and adapt to changing immune demands.

## Metabolism and function

Age-related alterations in cellular function arise from multiple underlying mechanisms, including genomic instability, telomere attrition and cellular senescence. More recently, it has been recognized that metabolic changes represent a key feature significantly impacting T-cell function [4].

In a nutshell, different T-cell subsets rely on distinct metabolic programmes to support their function. Naïve T cells mainly use oxidative phosphorylation to meet basal energy demands, while activated effector T cells increase aerobic glycolysis to support rapid proliferation and function. In contrast, memory and regulatory T cells rely on mitochondrial oxidative phosphorylation and fatty acid oxidation for long-term maintenance and immune regulation. Mitochondria play a dynamic role in intracellular signalling pathways. They can generate cues (such as reactive oxygen species, metabolites, calcium, and mitochondrial nucleic acids/peptides) and integrate inputs from nutrient availability, stress, and various immune pathways to coordinate cell fate and function [5, 6]. Importantly, mitochondria provide energy required for T-cell activation, proliferation, effector responses, and cellular longevity. For example, CD4+ CAR-T cells detected a decade after original infusion showed a profile reliant on mitochondrial metabolism [7]. Over time, mitochondria age—characterized by overall reduced function but increased production of damaging reactive oxygen species. These mitochondrial defects contribute, and likely cause many of the features seen in overall ageing of the individual as well as that of T cells (reviewed in depth by [8, 9]).

The T-cell compartment undergoes significant alterations over the life course. Both CD4+ and CD8+ naive T-cell numbers decline. Defects in autophagy and mitochondrial-maintained redox balance have been shown to skew differentiation of CD4+ helper T cells towards IL-17, IL-21-producing cells [10, 11]. Terminally differentiated CD8+ effector memory T cells characterized by the re-expression of the common leukocyte antigen CD45RA (T_EMRA_), are particularly susceptible to metabolic decline, exhibiting reduced mitochondrial fitness and increased oxidative stress. Despite this mitochondrial impairment, senescent CD8+ T_EMRA_ cells show elevated capacity for production of inflammatory cytokines, likely contributing to the increased low-grade inflammation in ageing termed inflammageing [11, 12]. As naive T cells decline, memory, effector and T_EMRA_ cells dominate with age, and the overall T-cell repertoire becomes increasingly narrow. This can hamper the response to new antigens impairing vaccination responses, as well as alter the immune response produced [13]. For further review on mechanisms of T-cell ageing, we refer the reader to recent publications [2, 4, 14].

Increasing age is a risk factor for tumour progression, which in turn suppresses T-cell function. The tumour microenvironment is characterized by nutrient and oxygen depletion as well as production of suppressive metabolites such as high levels of lactate [15]. Here, tumour cells outcompete and suppress effector T cells through metabolic inhibition. The reduced metabolic resilience of aged T cells likely compromises their ability to mount effective anti-tumour responses more profoundly. While this interpretation is supported by the known metabolic features of aged T cells and the metabolic challenges imposed by tumours, further studies are needed to assess the exact mechanisms involved, thereby enabling targeted therapeutic intervention.

## Importance for cell therapies

Cancer is a leading cause of death in the aged population and the leading cause of death overall in the UK (ourworldindata.org). Immunotherapy has quickly become a promising treatment option, with chimeric antigen receptor (CAR)-T-cell therapy demonstrating remarkable success in treating haematological malignancies. While chronological age does not directly predict response to CAR-T cell therapy, older patients often present with more co-morbidities and frailty, which increases the probability of treatment-related complications [16–18]. These factors potentially exclude older patients from accessing and/or fully benefiting from effective T-cell-based treatments. Furthermore, the clinical impact of age-related T-cell metabolic changes and functional decline remains to be comprehensively studied/defined.

Delivering CAR-T cell therapy is a complex and resource-intensive process. Patients offered CAR-T treatment have typically exhausted multiple prior lines of therapy or experienced disease relapse. Following the collection of patient blood to obtain the patient’s T cells, these are genetically modified to express a chimeric antigen receptor (CAR) and need to be expanded to generate sufficient CAR-T cell numbers for treatment. Manufacturing can take several weeks and is often performed at specialized off-site facilities, necessitating cryopreservation of cellular material [19]. During this period, patients frequently require bridging therapies, including chemotherapy, radiotherapy, targeted therapies, or combinations thereof, to control disease progression until CAR-T cell infusion. In addition, all patients currently undergo lymphodepleting chemotherapy prior to CAR-T cell infusion to promote expansion and activity *in vivo* [19]. Since increasing patient age is associated with physiological changes in renal and hepatic function and increased frailty, this can reduce tolerance to these lines of treatment [20].

A major patient group to receive CAR-T cell therapy is patients with diffuse large B-cell lymphoma (DLBCL), the most common form of lymphoma in adults. Patients are, on average, 60–65 years of age at diagnosis, which increases their risk of disease relapse meaning these are the patients primarily in need of effective immune therapies [21]. Given how heavily pre-treated patients generally will be at the time of blood collection, the starting population of T cells likely has significantly reduced fitness and is suboptimal compared to cells that would come from a healthy individual. Importantly, we need to consider that age-associated decline in T cells adds another layer of difficulty to this already complex situation. This raises the question: What quality of product is it possible to achieve with a given patient-derived T-cell population?

Independent of chronological age, T-cell exhaustion and senescent phenotypes are associated with poorer response to CAR-T cell therapy [22, 23]. Conversely, an enrichment of less differentiated and ‘stem-like’ populations within the CAR product have been indicated to be associated with increased persistence following infusion. Aged tumour-specific T cells in mice have been shown to have lower transduction efficiency, enrichment of CD62L-CD44^hi^ effector-like cells compared to CD62L+CD44^hi^ central memory-like cells, and impaired tumour-cell killing *in vivo.* High levels of terminal exhaustion within the aged cells, driven by a loss of EPAS1 (HIF-2α) is a key mechanism contributing to this impaired persistence [24]. Furthermore, greater metabolic health and plasticity of CAR-T cells will support sustained effector function essential for T cells to ultimately eliminate the cancer [25–27]. Thus, CAR products will require a combination of both effector and memory stem-like cells to hit two goals: acute tumour removal and sustained control. Achieving this balance appears increasingly challenging when manufacturing CAR-T cells from older patients, whose circulating T-cell compartments often contain fewer undifferentiated and possibly stem-like populations. Current approaches are testing various strategies to drive desired T-cell differentiation, modulate metabolic pathways, improve manufacturing protocols and design novel CAR constructs with the aim to enrich the CAR product for ‘optimal’ T-cell differentiation features [28–31]. However, many of these approaches are developed and tested using healthy donor T cells, often from young individuals between 20 and 30 years of age. If CAR-T therapies are ultimately intended for older, heavily pre-treated patients, it is worth questioning whether findings derived from younger adult immune systems accurately reflect the biology of the target population. Reliance on this benchmark may overestimate what is biologically achievable and may miss therapeutic strategies which could be more beneficial in the patients most likely to require these therapies.

## The methodological blind spot

Integrating knowledge of T-cell ageing into the design, development, and evaluation of CAR-T cell therapies has the potential to improve not only outcomes for older patients but the overall success of the field. T cell exhaustion and senescence are not exclusive to older individuals, and many younger patients also fail to respond to cellular therapies. The reasons for this are undoubtedly complex and multifactorial. Nevertheless, if we want to maximize therapeutic potential, considerations of T-cell fitness should be built into the research process from the outset rather than addressed only at later stages of development. Regardless of age and perhaps especially in older patients, CAR-T cells are generated from some of the most challenged immune cells encountered in clinical medicine. If we do not strive to apply similar biological challenges to the experimental systems used to model and optimize these therapies, we risk overlooking limitations that become apparent only in the intended patient population. This may be one factor contributing to the persistent gap between preclinical promise and clinical success [32, 33].

Young, healthy donor T cells are, for many labs, including our own, obtained from willing individuals from the academic population, generously enabling research on primary human samples. Generally, young healthy cells ‘behave well’, that is, proliferate, survive, and function reproducibly in cell culture. However, placing these cells as the ‘normal’ or ‘baseline’ can be biologically misleading for immunotherapy research. For instance, if a manipulation improves function relative to a ‘young donor baseline’, that is encouraging, but it does not necessarily result in the same intervention restoring a patient-relevant phenotype. Conversely, a mediocre improvement compared with T cells from young controls may still be clinically meaningful when assessed against age-matched or treatment-exposed older comparators.

## Future perspectives

This is not to dismiss young, healthy donor samples, which are indispensable for mechanistic and fundamental T-cell work. Instead, we argue that age distribution should be intentional and not incidental. There is also a translational risk: if age-related limitations are only recognized after therapeutic platforms have been optimized in youthful donor cells, the gap between pre-clinical promise and actual clinical performance becomes harder to bridge.

There are several practical points to be considered:

First, incorporating, age-aware experimental design early in the pipeline. Choosing relevant controls/study participants will increase our understanding of cellular function over the life-course and likely result in more efficacious treatment approaches. Age-matching controls and experimental samples to the intended clinical cohort should become a routine consideration throughout the research pipeline, and especially for translational studies intended to develop therapies for predominantly older patients.

Second, donor characteristics and experimental design should be reported clearly and consistently. The field would benefit from consensus reporting standards, analogous to existing guidelines in other areas of biomedical research. Journals should mandate transparent reporting of individual donor characteristics (including but not limited to age), biological, experimental, and technical repeats. This will ensure that results can be interpreted more accurately and compared between independent studies. Human immunology is inherently variable, influenced by donor age, ethnicity, sex, environmental exposures and prior disease history. In this context, studies undertaken with small numbers of donors (i.e. the ubiquitous n of 3) should be interpreted cautiously. These can provide interesting observations in line with case reports, or generation of hypothesis, but might not provide the robustness to establish a new paradigm, mechanism, or generalized therapeutic strategy.

Third, investment to develop advanced experimental systems is needed. Murine models are indispensable for understanding aspects of immunology in a living mammal but cannot fully represent the vastly different exposures human immune systems experience over an 80+ year time course. Especially given that most animal facilities will keep genetically inbred animals, with severely limited microbiota, under specific pathogen-free conditions educating an immune system distinct from free-living mammals [34]. Consequently, better human-centric models need to be developed. The authors would here additionally like to note that the cessation of reproductive capacity (menopause) followed by many more years of life-expectancy in human females is a feature only shared with a small group of other mammals such as toothed whales and chimpanzees which make unsuitable laboratory models. Despite the fundamental impact menopause has on the immune system, this area remains woefully understudied. Improved *ex vivo* and *in vitro* systems to efficiently study human immunology and metabolism over the life course are required and need to be supported by ethical and equitable access to human samples, state-of-the-art biobanking, advanced (fluidic) organoid and tissue models, and single-cell approaches to study rare populations such as patient-derived CAR-T cells *ex vivo*. Vital for metabolic studies will be the development of improved ‘physiological culture conditions’ which, for example, take into account nutrient composition of media and oxygen tension [35].

Finally, it should be appreciated that physiological age does not necessarily match chronological age. Two people of the same chronological age can have vastly different physical health profiles. Ambitiously, the future direction would be to develop novel assays specifically designed to detect and quantify age-induced functional differences, so that properties of ageing can be measured accurately. Many other confounding factors, like sex, ethnicity and socio-economic background, contribute to the complexity of designing and improving treatments for a widely diverse population and must be considered more stringently in future research.

## Conclusion

If we are serious about considering the relevance of T-cell biology to immunotherapy, then age and other factors must be treated as biological variables. The current reliance on young, healthy donor cells and *in vivo* models risks embedding a systematic bias into the way we define T-cell fitness, persistence, differentiation, and therapeutic potential. A more age-aware research framework would not only sharpen our understanding of T-cell immunobiology but also improve the likelihood that preclinical findings translate into meaningful clinical benefit for the populations who need these therapies most. The challenge is not simply to acknowledge the age gap, but to close it at the point where experimental questions are first asked.

## Data Availability

Not applicable.

## Permission to reproduce

Figure created in BioRender. George, M. (2026) https://BioRender.com/rvho24h.

## Abbreviations:

CAR: chimeric antigen receptor
DLBCL: diffuse large B-cell lymphoma
EPAS1: endothelial PAS domain-containing protein 1
HIF-2α: hypoxia-inducible factor 2-alpha
T_EMRA_: effector memory T cells that re-express CD45RA
